# Ethnomedicinal Plants Traditionally Used for the Treatment of Jaundice (Icterus) in Himachal Pradesh in Western Himalaya—A Review

**DOI:** 10.3390/plants10020232

**Published:** 2021-01-25

**Authors:** Disha Raghuvanshi, Rajni Dhalaria, Anjali Sharma, Dinesh Kumar, Harsh Kumar, Martin Valis, Kamil Kuča, Rachna Verma, Sunil Puri

**Affiliations:** 1 School of Biological and Environmental Sciences, Shoolini University of Biotechnology and Management Sciences, Solan 173229, India; disha.raghuvanshi786@gmail.com (D.R.); rajnidhalaria86@gmail.com (R.D.); anjalisharmaas8749347@gmail.com (A.S.); spuri56@yahoo.com (S.P.); 2 School of Bioengineering and Food Technology, Shoolini University of Biotechnology and Management Sciences, Solan 173229, India; dkchatanta@gmail.com (D.K.); microharshs@gmail.com (H.K.); 3 Department of Neurology of the Medical Faculty of Charles University and University Hospital in Hradec Kralove, Sokolska 581, 50005 Hradec Kralove, Czech Republic; martin.valis@fnhk.cz; 4 Department of Chemistry, Faculty of Science, University of Hradec Kralove, 50003 Hradec Kralove, Czech Republic; 5 Biomedical Research Center, University Hospital in Hradec Kralove, Sokolska 581, 50005 Hradec Kralove, Czech Republic

**Keywords:** jaundice, bilirubin, ethnomedicinal plants, phytoconstituents, hepatoprotective

## Abstract

Ethnomedicinal plants have a significant role in the lives of people of rural and tribal areas. Thousands of medicinal plant species are used to treat various diseases, including jaundice, and are considered an important therapeutic resource to minimize these diseases. Jaundice (icterus) is a chronic disease that occurs when the amount of bilirubin in the blood increases. This review describes different ethnomedicinal plants used for curing jaundice by tribal and rural people of Himachal Pradesh. The study reveals 87 ethnomedicinal plant species belonging to 51 different families, which are used for treating jaundice in Himachal Pradesh. These plants are arranged in a systematic way, which includes a description of their common name, botanical name, along with its family, plant parts used, region, and mode of use in tabulated form. Some of the plant extracts have already been explored for their phytochemical and pharmacological significance and proved their potential in the preparation of new medicines or drugs against the treatment of jaundice. This review is an attempt to highlight the indigenous knowledge of medicinal plants, which are specifically used for the treatment of jaundice. The data mentioned in the present review is compiled from various sources like existing literature, books, Google Scholar, and Scopus publications. Among all the observed plant species, most used medicinal plants for the treatment of jaundice include *Justicia adhatoda*, *Emblica officinalis*, *Ricinus communis*, *Saccharum officinarum*, *Terminalia chebula*, *Berberis aristata*, *Cuscuta reflexa,* and *Tinospora cordifolia.* Plants that are mostly utilized for the treatment of jaundice need to be scientifically validated by pharmacological analysis and should be subsequently used for the preparation of new drugs, which may prove far more beneficial than the existing one.

## 1. Introduction

Himachal Pradesh is one of the Himalayan states of India, which has been regarded as the richest resource of biodiversity. The area is rich in medicinal plants, which are widely used by the people of different tribal and rural areas. The state is divided into 12 districts and 169 tehsils and sub-tehsils, covering a total area of 55,673 km^2^ with altitude variation from 200–7000 m above sea level [1]. Geographically, the state shows three different zones or regions, known as the outer Himalaya, mid-hills, and the greater Himalaya. The outer Himalaya, also known as the Shiwalik hills, ranges from 350 m to 1500 m above sea level and includes districts, such as Bilaspur, Sirmour, Kangra, Una, Chamba, Mandi, Hamirpur, and some parts of Solan. Mid-hills (1500 to 3500 m above sea level) cover the area of Mandi, Kangra, Chamba, Solan, Shimla, Lahaul-Spiti, Kinnaur, and Kullu districts. The Greater Himalaya (ranges 3500 and above) covers the area of Lahaul-Spiti, Kullu, and Pangi tehsil of Chamba district, which is also known as the high-altitude alpine area [2,3]. Himachal Pradesh in the lap of western Himalaya is well-known for its floral diversity, including medicinal plants, which are used since ancient times for the treatment of jaundice. The dependency of human beings on plants is an age-old relationship, which is described as ‘ethnobotany’. Ethnobotany comes from the term ethnology, which means the study of culture, so ethnobotany or ethnobiology is a scientific study of plants and human relationship, which shows plants as a primary source of need. Ethnobotany deals with various aspects in which one of the most popular and common aspect is the study and use of ethnomedicines. Ethnomedicine involves the study of indigenous beliefs, concepts, knowledge, and practices among the ethnic groups of tribal and rural people for preventing, curing, and treating jaundice. For human existence, since ancient times, especially, the ethnic or tribal community has a great dependence on local flora for medicinal and other purposes [1,4]. The traditional medicine system represents the indigenous beliefs, skills, and practices of rural and tribal communities based on their experiences to maintain their health [5]. Traditional medicines play an efficient role in the preparation of herbal drugs for the betterment of people [6]. This system of medicines is used for curing diseases through the employment of agencies and forces of nature. Tribal people have their own system of medicines, which are age-old, and some of which are not documented in the literature. This tradition has been passed on from one generation to the other for treating jaundice. The information on medicinal and various other plants comes from the ancient people when they started learning and making use of these traditional plants for various purposes [7,8].

Medicinal plants are regarded as the gift of nature to humans. Various parts of medicinal plants, including herbs, shrubs, and trees, are used for curing jaundice and diseases like neurodegenerative, inflammatory, anthelmintic, diaphoretic, diuretic, etc. According to WHO (World Health Organization), “medicinal plant is a plant, within which one or more of its part contains the substances, which can be further used for various therapeutic purposes, and serves as a precursor for chemo-pharmaceutical semi-synthesis” [9]. Various bioactive compounds of plants called the secondary metabolites are the reason for their medicinal value and include glycosides, tannins, steroids, alkaloids, terpenoids, essential oils, etc. [10]. Himachal Pradesh is endowed with a rich diversity of plants, which includes 3500 higher plants, and of these, 1500 plants are identified with medicinal and aromatic properties [11]. Because of the geographical position and difficult means of transport and communication, people of some major tribes of Himachal Pradesh (Gaddi, Gujjar, Kinnaura, Lahula, and Pangwals) mostly live in villages and rural areas and belongs to diverse cultures. These people, with their specific traditional knowledge, make use of different medicinal plants for curing jaundice [1]. Medicinal and ethnobotanical uses of different plant species are documented by various researchers or scientists from different areas of Himachal Pradesh based upon the information provided by the local ethnic people [12]. Ethnomedicines have made good contributions in the health care system in traditional medicines for the treatment of jaundice since ancient times. There are two broad categories for the use of medicinal plants; firstly, plants are used traditionally only by local physicians for getting relief from illness, and secondly, the plants are used by pharmaceutical companies for their active ingredients [13]. According to WHO, due to poverty and lack of modern medicines among different rural and tribal areas, it is estimated that about 70–80% of the world’s total population is totally dependent on the local medicinal plants for their primary healthcare system [8]. 

**Figure 1:**
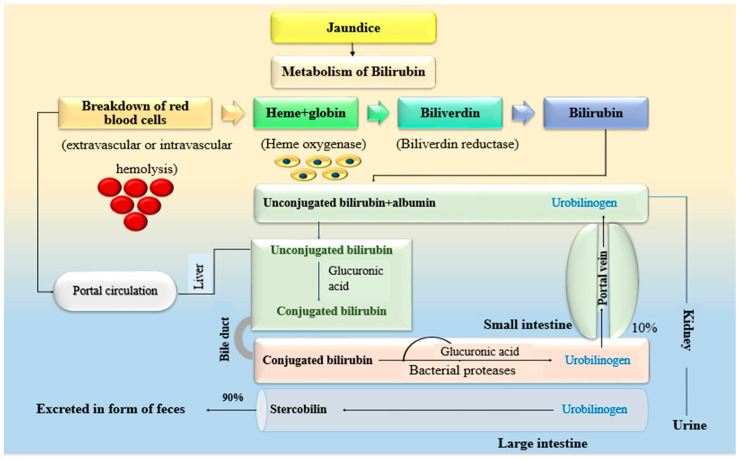
Bilirubin metabolism or pathophysiology of jaundice [15].

Ethnomedicinal plants are generally used for curing various ailments like diabetes, dysentery, typhoid, and jaundice. Different parts of the plant, including roots, leaves, fruits, and flowers, are used for the treatment of jaundice. Furthermore, jaundice is not just a disease rather a sign of a disease that occurs in the liver, which indicates impairment of the liver functioning [14,15]. The foremost ancient literature says that “iecur” is a Latin word that was previously used to describe the term liver [16]. Basically, the term jaundice is taken from the french word “jaune”, which means “yellowness” and is characterized by yellow pigmentation [17]. Pigmentation is generally shown by the skin and eyes. It occurs due to the exceeding level of bilirubin. Bilirubin is synthesized in the body and is a natural product that is produced because of hemolysis through the action of liver cells, which further in the presence of biliverdin reductase leads to the production of bilirubin or unconjugated bilirubin. The metabolism of bilirubin describes the pathophysiology of jaundice, as shown in Figure 1. 

Under normal circumstances, unconjugated bilirubin (lipid-soluble) in the presence of glucuronic acid gets converted into conjugated bilirubin (water-soluble), which is released in the small intestine. Removal of glucuronic acid in the presence of bacterial protease takes place in the small intestine, which further passes through the large intestine in the form of feces, and the remaining enters the kidney by the portal vein and passes out as urine, as shown in Figure 1 [15]. When this bilirubin accumulates in the blood, skin, sclera, and mucous membrane, it turns yellow in color. This yellowness of skin and other parts is generally called jaundice or icterus and is usually seen when the amount of plasma bilirubin is greater than its normal value, i.e., 2 mg/dL [18]. 

According to the pathophysiology of jaundice, it is mainly caused due to increased level of bilirubin and its overproduction in the liver, which may occur due to many reasons like acute or minor liver inflammation, obstruction of the bile duct, Gilbert’s syndrome, cholestasis, and hemolytic anemia [15]. Jaundice is usually found much more effective and serious in adults rather than in new-born children (neonates), and sometimes it causes even death of the adult individual [19].

**Figure 2:**
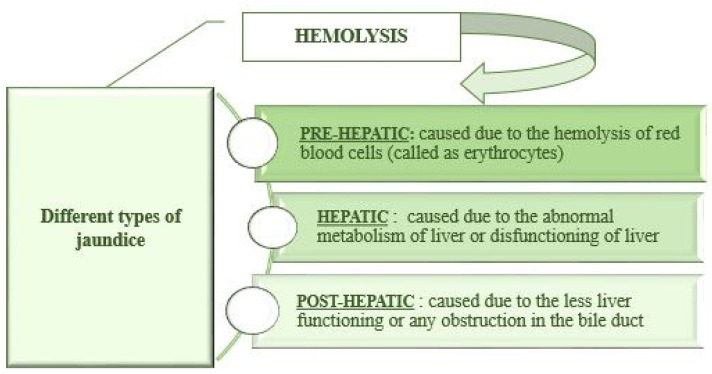
Types of jaundice according to its pathophysiology [15].

Jaundice shows three different stages or types based on its pathophysiology: pre-hepatic jaundice that is caused due to the hemolysis of red blood cells, also called erythrocytes. Hepatic jaundice occurs due to the abnormal metabolism of the liver or dysfunction of the liver, and post-hepatic jaundice is caused due to less liver functioning or any obstruction in the bile duct, as described in Figure 2.

Jaundice can also be a viral disease, which can spread through contaminated water and food-related items or due to poor sanitization conditions or through several other diseases, such as hepatitis A, hepatitis B, hepatitis C, hepatitis D, liver cancer, and hemolytic anemia, etc., which damage liver [14]. It has been estimated that more than two billion people worldwide are infected annually with the hepatitis B virus [20]. The history of jaundice is very long and shown in ancient Ayurveda and the Indian traditional system of medicines [21]. Jaundice is also known as Hariman disease in Rigveda (8000 BC). Herbal treatment is prescribed for jaundice because medicinal plants show a faster rate of reduction in cases when compared with western medicines [22]. This disease shows different kinds of symptoms like weakness, high fever, nausea, loss of appetite, vomiting, and the main symptom shown by this disease is the dark urine color. Sometimes, it also leads to serious conditions like coma, a sudden attack of illness or epileptic fits, psychosis (like having a severe mental disorder), and finally, death of the patient. Precautions or prevention for jaundice generally requires a low-fat diet, high water intake as much body requires, and mainly a healthy diet routine and proper nutrition [15].

Due to cultural or historical reasons and the high cost and side effects of allopathic medicines, traditional and herbal medicines have gained popularity for curing jaundice. So, numerous ethnomedicinal plants have been used by the people of different tribes and communities for treating jaundice based on their indigenous knowledge. Thus, this review is an attempt for the exploration of ethnomedicinal plants used for the treatment of jaundice, which can be cured by locally available plants or with the help of hermits. 

## 2. Materials and Methods

All information regarding plants was collected through published data, including the botanical name of plants, family name, part used, and mode of use. The number of articles reviewed were available as published work on online databases (Science Direct, Pubmed, Web of Science, and Google Scholar) and were found using different key phrases (jaundice, ethnomedicinal plants, Himachal Pradesh, traditional uses, western Himalaya, and biological activity). The present study was revised from different scientific articles, including 108 research papers, 30 review papers, and 6 books from 1970 to the 2020 year. Botanical names of different plant species were validated from the online website (www.theplantlist.org).

### 2.1. Ethnomedicinal Plants Used in the Treatment of Jaundice in Himachal Pradesh

It was observed that approximately 87 ethnomedicinal plants are used by the tribal and rural communities of Himachal Pradesh for curing jaundice, and this information is described in Table 1, where plant families are arranged in alphabetical order and include plant’s botanical name, common name, family, and region (where these plants were reported).

**Figure 3:**
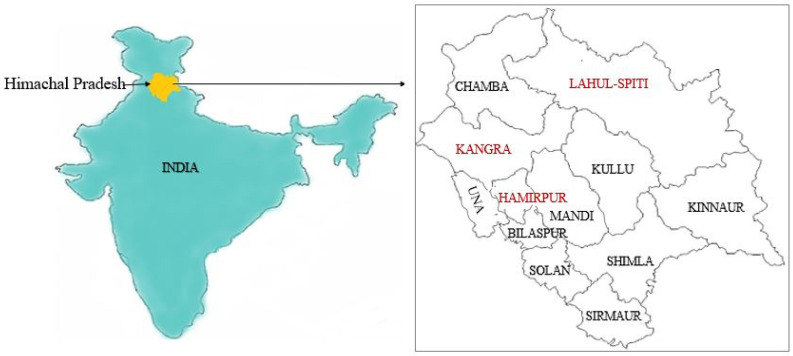
Map of India, showing districts of Himachal Pradesh (district names are added in red font where maximum number of medicinal plants were reported for jaundice treatment).

The study revealed that these 87 ethnomedicinal plants show variations among them and represent 51 different families of plants used for the treatment of jaundice in Himachal Pradesh. Most of these plant species used for treating jaundice were observed in different areas of Himachal Pradesh, including different districts, such as Kangra, Hamirpur, and Lahul and Spiti, as shown below in Figure 3. Among all these plant species, common medicinal plants belong to six major families, i.e., Asteraceae, Fabaceae, Euphorbiaceae, Gentianaceae, Lamiaceae, and Solanaceae. 

**Figure 4:**
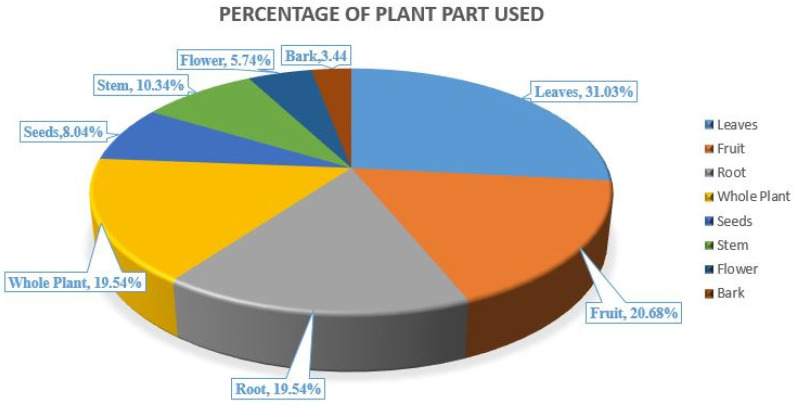
Analysis of the data showing a total estimation of plant parts used for the treatment of jaundice [8,44].

The plant species most frequently used for the treatment of jaundice include *Justicia adhatoda*, *Emblica officinalis*, *Ricinus communis*, *Saccharum officinarum*, *Terminalia chebula*, *Berberis aristata*, *Cuscuta reflexa*, and *Tinospora cordifolia*, and this shows the richness of information regarding medicinal plants used by the people of Himachal Pradesh. Medicinal plants like *Aloe vera* contains many bioactive compounds, which are responsible for many medicinal properties, such as antibacterial, antioxidants, and immunity-boosting property [18]. Various plant parts have been used for curing jaundice, including stem, leaves, roots, bark, fruits, flowers, seeds, and sometimes even whole plant (described in Table 2). This also strengthens the use of these plants by local people. All ethnomedicinal plants contain some phytochemical constituents, which may be effective in showing an impact on the disease and its cure. The ethnomedicinal significance of plants has been proved by a reassessment of their efficiency potential in different regions. In all the reported plants, there is variation in the usage of plant parts for treating jaundice, which is also shown in Figure 4. This is similar to the work performed in south-western Nigeria, which shows the data analysis of different plant parts used for the treatment of various ailments, including jaundice [8,43,44]. 

### 2.2. Mode of Plant Used for the Treatment of Jaundice in Himachal Pradesh

Traditionally, people of Himachal Pradesh use these plant species in appropriate dosages or amounts for curing jaundice. There are different methods of usage of these medicinal plants, like in district Hamirpur, bulb of *Colocasia esculenta* is cooked as a vegetable, the dried bulb is cut into pieces and then crushed to make powder and given orally to treat jaundice, and juice of fresh *Pistacia integerrima* is given daily to cure jaundice [24]. Uses of other ethnomedicinal plants are described in Table 2, which includes the plant’s botanical name, plant part used, and mode of use (route of administration) of these plants for treating jaundice. In most cases, these plants are taken alone as a decoction or with a combination of the talmishri or kali mirch. On the other hand, some plants are also taken in combination with other plants, e.g., *Emblica officinalis* fruit is taken along with the *Terminalia chebula* and *Terminalia bellirica* fruits, grounded into powder form, and taken orally to cure jaundice [25].

The above pie-chart highlights the different plant parts commonly used for the treatment of jaundice, as analyzed from data in Table 2. It was observed that for jaundice treatment, leaves are highly utilized (31.03%), followed by fruits (20.68%), roots (19.54%), whole plant (19.54%), seeds (8.04%), stem (10.34%), flowers (5.74%), and bark (3.44%); the same pattern is also shown in Figure 3. This indicates that in most cases, the leaves of medicinal plants have more significance than any other plant part. Hence, it can be concluded that leaves are highly effective for curing jaundice, which may be due to more phytochemical accumulation in the plant leaves. However, fruits are the second most used to treat jaundice, root belongs to the third position for treating jaundice, and bark is utilized in the least cases.

Intake of these ethnomedicinal plant parts is suggested to be continued for a definite period or until full recovery. Thus, plants are suggested to be taken in the form of paste, decoction, extract, and dried powder form. Different plant parts contain various organic compounds within them, called secondary metabolites, which may be the reason behind the effectiveness of plant-based treatments and show various chemical and physiological actions against jaundice. These phytoconstituents include proteins, carbohydrates, steroids, terpenoids, alkaloids, saponins, phenols, flavonoids, vitamins, tannins, and essential oils, which show an inhibitory effect against hepatoprotective diseases, mainly against jaundice [45,46]. *T. chebula* contains a significant amount of phenolic and flavonoid compounds. The main constituents of the plant include 2,4-chebulyl-β-D-glucopyranose, ellagic acid, gallic acid, and chebulic ellagitannins. *T. chebula* is one of the main components of the important Ayurvedic formulation “Triphala” (infusion of three fruits, i.e., *T. chebula*, *T. bellirica,* and *Emblica officinalis*). Ayurveda prescribes this formulation to cure kidney and liver dysfunctions. *T. chebula* extract ensures hepatoprotection against liver diseases, such as jaundice, due to its antioxidant activity and bilirubin level lowering effect. The reduction in serum bilirubin level is the most important evidence, which supports the traditional use of the plant against jaundice [47].

### 2.3. Phytochemical Constituents Present in the Ethnomedicinal Plants

The medicinal property of plants is mainly because of the formation or stimulation of various chemical compounds that occur naturally in the plants and hence used to cure jaundice and various other diseases. So, this review describes various phytochemical constituents present in the ethnomedicinal plants used by people of Himachal Pradesh for curing jaundice, and the data is taken from different sources. The plants are further used for the development of antimicrobial and antioxidant drugs [48], thus proving their medicinal worth. Plant like *Justicia adhatoda* consists of various organic [49] and bioactive compounds [50], which possess numerous biological activities, such as antitussive, abortifacient, cardiovascular protection, anti-inflammatory, and antimicrobial. *Berberis lycium* plant shows the presence of tannins, terpenoids, fats, resins, and many active alkaloids. The roots are the foremost important part of the *Berberis* species as they contain a variety of alkaloids, and the most prominent one is berberine. It has been found that the inhibitory activity of *Berberis lycium* is shown by the components present in its root extract [51]. Like this, many other plants are used in the treatment of jaundice, possessing various phytochemical constituents, out of which some predominant phytochemicals are described in Table 3. 

Ethnomedicinal documentation combined with the screening of various biological properties of plants is one of the convincing ways of discovering new drugs against drug-resistant pathogens in the modern era or against the diseases related to oxidative stress, including jaundice. The effectiveness of these phytochemicals used in treating jaundice and other diseases has been seen in their biological activities, such as antimicrobial, antibacterial, antitumor, antiviral, or antioxidant activity. The oxidative stress of free radicals is directly associated with the presence of pathogenic organisms or due to disease-causing mechanisms of different ailments like cancer, diabetes, and inflammatory diseases [117].

### 2.4. Ethnopharmacological Evidence of Some Plant Species Used for the Treatment of Jaundice

As described in Table 3, the beneficial effects of medicinal plants are due to the presence of different bioactive compounds that are responsible for the treatment of jaundice. Ethnomedicinal studies combined with phytochemicals are one of the convincing approaches for ethnopharmacological studies [118]. The medicinal effect of different plant parts shows various hepatoprotective activities, including the curing of various liver diseases in which one of the major diseases is jaundice. Various ethnomedicinal plants are traditionally used for the treatment of jaundice, while some plants promote the discovery of active compounds, which further aids in the development of synthetic drugs against jaundice. Although some epidemiological studies are required for the practical implementation of the plants for jaundice treatment [47]. To assess the ethnomedicinal significance of the hepatoprotective plants used, particularly for treating jaundice, different plant species have been reported to be used in various in vivo experiments (Table 4). Table 4 includes various plant species, plant parts used/extracts taken, toxicant and its dose, experimental model (the animal model used for study), constituents that may be responsible for hepatoprotective activities, and their effectiveness against jaundice [15]. 

In vivo experimental studies with these plants (Table 4) have shown effective results in the treatment of jaundice and confer scientific evidence regarding plant use in the same. In most of the in vivo studies, hepatotoxicity is introduced with CCl_4_ or with paracetamol; however, in few cases, gentamycin, thioacetamide, t-BHP, and ethanol are also used for the same. Phytochemicals observed for curing hepatotoxicity are phenolic and flavonoid compounds as a major factor in curing hepatotoxicity in most of the in vivo studies. In some cases, hepatotoxicity is reduced by the decrease in serum bilirubin level and an increase in the antioxidant defense system.

As few plants are evaluated with their experimental studies, so this study needs to be intensified more on those plant extracts, which are used extensively for jaundice treatment. Further, nano-formulation of plant extracts enhances their medicinal significance [143,144], so nano-formulation of herbal plants can also be used as an alternative for curing jaundice in the future. At the same time, farmers should be encouraged for the commercial production of important medicinal plants and should further have support from industry and government. 

## 3. Conclusions

Ethnomedicinal knowledge is respected by rural people and has been shown to be useful in the treatment of various diseases and the production of medicines in the Western Himalaya from time to time. Traditional or folk-based plant medicines have shown great potential to form the basis of jaundice-curing drugs. The purpose of the present study was to record the ethnomedicinal knowledge of plants used for the treatment of jaundice by the rural and tribal communities of Himachal Pradesh in western Himalaya. The other aims of this research were to discuss the different important phytochemicals and active compounds present in these plants and to discuss the different in vivo studies performed in support of their medicinal uses, with specific reference to the treatment of jaundice. The outcome of this research showed that the rural people of Himachal Pradesh used 87 different plant species with 51 different families to treat jaundice and contribute to healthcare. These plants demonstrated the presence of several phytochemicals in them and displayed phenolic and flavonoid compounds with hepatoprotective properties in most of the experimental studies (in vivo) performed with these plants. With antioxidant potential, the phenolic and flavonoid compounds are recognized, and due to this property, these plants have been shown to be important in curing jaundice. *Aloe vera*, *Bauhinia variegata*, *Berberis aristata*, *Emblica officinalis,* and *Terminalia chebula* are some of these herbs, which suggest the ethnopharmacological approach to treating jaundice with the hepatoprotective operation.

There is a lot of knowledge in the latest literature on the use of various plants for treating jaundice. Nevertheless, very few studies are carried out on the scientific validation of medicinal plants by means of biochemical, clinical, and pharmacological screening to validate the jaundice healing folklore medicine. In the future, it is, therefore, very important to pursue steps that do not deviate from shifting the view of tribal people toward their indigenous belief in the treatment of jaundice to develop successful drugs or to discover new potential sources of drugs. In addition, nano-formulation of plant extracts also improves their therapeutic significance [143,144], and it is also possible to use nano-formulation of herbal plants as an alternative and refining conventional knowledge for the potential cure of jaundice.

## Figures and Tables

**Table 1:** Ethnomedicinal plants used to cure jaundice by traditional healers in Himachal Pradesh.

Botanical Name	Local Name	Family	Region	Data Source
*Justicia adhatoda* L.	Basuti, Arusha	Acanthaceae	Kangra, Hamirpur	[23,24]
*Adiantum capillus-veneris* L.	Dooman tuli	Adiantaceae	Hamirpur	[24]
*Pistacia integerrima* J. L. Stewart ex Brandis	Kakar Singhi	Anacardiaceae	Hamirpur	[24]
*Carissa opaca* Stapf. ex Haines.	Karaunda, Garnu	Apocynaceae	Kangra, Mandi, and Una	[25]
*Calotropis procera* (Aiton) Dryand.	Aak	Apocynaceae	Kangra and Chamba	[26]
*Colocasia antiquorum* Schott	Ghandiale, Arbi	Araceae	Kangra	[23]
*Colocasia esculanata* (L.) Schott	Arbi kuchawari	Araceae	Hamirpur	[24]
*Hedera helix* L.	Kannauri (Bail)	Araliaceae	Shimla	[27]
*Ajania tibetica* (Hook.f. and Thomson) Tzvelev	Tibetan Tansy	Asteraceae	Lahul and Spiti	[28]
*Crepis flexuosa* (Ledeb.) Benth. ex C.B. Clarke	Homa-silli	Asteraceae	Lahul and Spiti	[28]
*Conyza japonica* (Thunb.) Less. ex Less.	Gaadi	Asteraceae	Kullu (Solang Valley)	[29]
*Picris hieracioides* subsp. japonica (Thunb.) Hand-Mazz.	Cherakpa	Asteraceae	Lahul and Spiti	[30]
*Scorzonera divaricata* Turcz.	Himalayan viper grass	Asteraceae	Lahul and Spiti	[31]
*Taraxacum officinale* (L.) Weber ex F.H. Wigg.	Dudhli, Dulal	Asteraceae	Kangra, Hamirpur	[23,24]
*Youngia tenuifolia* (Willd.) Babc. and Stebbins	Seertik	Asteraceae	Lahul and Spiti	[28]
*Vernonia anthelmintica* (L.) Willd.	Kaliziri	Asteraceae	Shimla	[27]
*Berberis aristata* DC.	Kashmal, Chunchari	Berberidaceae	Kangra, Mandi, Shimla, Chamba	[23,27,32,33]
*Berberis ceratophylla* G. Don	Kashmal	Berberidaceae	Kangra	[34]
*Berberis lycium* Royle	Kashmal, Dauhaldi	Berberidaceae	Kangra, Hamirpur, Shimla, Chamba, and Sirmour	[23,24,27,32,35]
*Berberis chitria* Buch. -Ham. ex. Lindl.	Kashmal	Berberidaceae	Shimla	[27]
*Betula utilis* D. Don	Bhojpatra	Betulaceae	Kangra and Chamba	[23,32]
*Capsella bursa-pastoris* (L.) Medik.	Jangli sarson	Brassicaceae	Kullu (Solang Valley)	[29]
*Raphanus sativus* L.	Muli	Brassicaceae	Kangra, Hamirpur	[23,24]
*Oroxylum indicum* (L.) Kurz	Tatpalnga	Bignoniaceae	Kangra	[23]
*Cassia fistula* L.	Kyar, Alsi ki tat	Caesalpinaceae	Kangra	[34]
*Tamarindus indica* L.	Imli	Caesalpinaceae	Hamirpur	[24]
*Capparis spinosa* L.	Kabra findus rose	Capparaceae	Lahul and Spiti	[31]
*Carica papaya* L.	Pump, Papita	Caricaceae	Kangra	[23]
*Terminalia bellirica* (Gaertn.) Roxb.	Behada	Combretaceae	Solan, Kangra	[23,36]
*Terminalia chebula* Retz.	Harad	Combretaceae	Kangra, Solan (Kunihar forest divison), and Shimla	[23,27,36]
*Cuscuta reflexa* Roxb.	Akash bel	Convalvulaceae	Hamirpur, Bilaspur, Solan (Kunihar forest divison)	[24,27,37]
*Cucumis sativus* L.	Kheera	Cucurbitaceae	Hamirpur	[24]
*Elaeagnus rhamnoides* (L.) A. Nelson	Sea-buck thorn	Elaegnaceae	Lahul and Spiti	[31]
*Hippophae tibetana* Schltdl.	Star bn	Elaegnaceae	Lahul and Spiti	[31]
*Emblica officinalis* Gaertn.	Amla, Amlika	Euphorbiaceae	Kangra, Shimla, Bilaspur, Chamba, Hamirpur, Sirmour, Solan, Una	[23,24,25,32,37,38]
*Euphorbia hirta* L.	Doodhli	Euphorbiaceae	Hamirpur	[24]
*Euphorbia tirucalli* L.	Tohar	Euphorbiaceae	Una, Hamirpur	[39]
*Mallotus philippinensis* Mull.Arg.	Kamla tree, Kumkum	Euphorbiaceae	Kunihar forest division, Solan	[36]
*Phyllanthus fraternus* G.L. Webster	Chota amla, Bhoomi ambla	Euphorbiaceae	Kangra	[23]
*Ricinus communis* L.	Erand	Euphorbiaceae	Kangra	[23]
*Equisetum arvense* L.	Girthan	Equisetaceae	Kangra	[23]
*Bauhinia variegata* L.	Karale, Kachnar	Fabaceae	Kangra	[23]
*Cajanus cajan* (L.) Millsp.	Arhar, Pigeonpea	Fabaceae	Chamba	[40]
*Cicer microphyllum* Benth.	Chana, Cowpea	Fabaceae	Lahul and Spiti	[28]
*Trigonella emodi* Benth.	Methi, Fenugreek	Fabaceae	Lahul and Spiti	[31]
*Flacourtia ramontchi* L.	Governor’s plum, Bilangra	Flacourtiaceae	Bilaspur, Chamba, Kangra, Hamirpur, Sirmour (Nahan), Solan, Una	[25]
*Gentiana kurrroo* Royle	Kanauri (Bail)	Gentianaceae	Shimla	[27]
*Gentiana tubiflora* (G. Don) Griseb.	Tikta anupo Mensa	Gentianaceae	Lahul and Spiti	[30]
*Gentiana leucomelaena* Maxim.	Buksuk shipo	Gentianaceae	Lahul and Spiti	[30]
*Gentianopsis detonsa* (Rottb.) Ma	Chateek	Gentianaceae	Lahul and Spiti	[30,31]
*Gentianopsis paludosa* (Hook.f.) Ma	Gyatheek	Gentianaceae	Lahul and Spiti	[30,31]
*Gentianella moorcroftiana* (Wall. ex Griseb.)	Airy shaw	Gentianaceae	Lahul and Spiti	[31]
*Geranium nepalense* Sw.	Tirahi	Geraniaceae	Manali	[26]
*Mentha spicata* L.	Pudina	Lamiaceae	Kangra	[23]
*Aloe vera* (L.) Burm.f.	Kware, Ghritkumar, Gavrapatha	Liliaceae	Kangra, Shimla	[23,38]
*Asparagus adscendens* Roxb.	Sanspan	Liliaceae	Kangra	[23]
*Woodfordia fruticosa* (L.) Kurz	Dhoaien, Dhai	Lythraceae	Kangra, Hamirpur	[23,24]
*Tinospora cordifolia* (Willd.) Miers	Giloe, Giloen, Guljae	Menispermaceae	Kangra, Chamba	[23,32]
*Morus alba* L.	Chitta toot	Moraceae	Hamirpur, Bilaspur	[24,37]
*Morus nigra* L.	Kala toot	Moraceae	Hamirpur	[24]
*Leucas cephalotes* (Roth) Spreng.	Mal bhedu	Lamiaceae	Kangra	[23]
*Boerhavia diffusa* L.	Punarnava	Nyctaginaceae	Una and Hamirpur	[39]
*Argemone mexicana* L.	Kantili, Pili Kantili, Bharbhand	Papaveraceae	Kangra, Hamirpur, and Sirmour	[23,24,35]
*Sesamum indicum* L.	Til	Pedaliaceae	Hamirpur	[24]
*Polygonum tortuosum* D. Don	Agel davaj	Polygonaceae	Lahul and Spiti	[30]
*Persicaria amplexicaulis* (D. Don) Ronse Decr.	Amli/kutrya	Polygonaceae	Chamba and Kangra	[26]
*Hordeum vulgare* L.	Jou, Joui	Poaceae	Hamirpur, Bilaspur	[24,37]
*Saccharum officinarum* L.	Ganna, Kamandi	Poaceae	Kangra, Bilaspur, and Hamirpur	[23,24,37]
*Podophyllum hexandrum* Royle	Bankakdi	Podophyllaceae	Manali	[41]
*Punica granatum* L.	Daran	Punicaceae	Hamirpur	[24]
*Aquilegia fragrans* Benth.	Zadul	Rannunculaceae	Kangra	[34]
*Aconitum rotundifolium* Kar. and Kir.	Atish, Patish	Rannunculaceae	Lahul and Spiti	[31]
*Thalictrum foliolosum* DC.	Pili jari, Chabra	Rannunculaceae	Shimla	[27]
*Geum elatum* Wall. ex G. Don	Gyampar mendok, Turu silva Mensa	Rosaceae	Lahul and Spiti	[30]
*Prunus domestica* L.	alubhukhara, Palam	Rosaceae	Hamirpur	[24]
*Rosa webbiana* Wall. ex Royle	Seba, Webb’s rose	Rosaceae	Lahul and Spiti	[31]
*Rubia manjith* Roxb. ex Fleming	Jamithi, Manjit	Rubiaceae	Manali	[42]
*Aegle marmelos* (L.) Correa	Bil, Bil patri	Rutaceae	Hamirpur, Bilaspur	[24,37]
*Saxifraga flagellaris* Willd.	Spider plant	Saxifragaceae	Lahul and Spiti	[28]
*Picrorhiza kurroa* Royl ex. Benth.	Karru, Kutki	Scrophulariaceae	Hamirpur, Manali, Chamba	[24,32,41]
*Capsicum annum* L.	Mircha, Pippali	Solanaceae	Kangra	[23]
*Datura stramonium* L.	Dhatura	Solanaceae	Manali	[42]
*Solanum nigrum* L.	Choote tamatter, Makoi	Solanaceae	Kangra, Hamirpur	[23,24]
*Solanum surattense* Burm. f.	Kantkari	Solanaceae	Hamirpur	[24]
*Centella asiatica* (L.) Urb.	Brahmi, Minki	Umblellifereae	Kangra	[23]
*Urtica dioica* L.	Bichu butti	Urticaceae	Manali	[42]
*Viola serpens* Wall. ex Ging.	Bhanaksha	Violaceae	Hamirpur	[24]

**Table 2:** Mode of use of ethnomedicinal plants for treating jaundice.

Botanical Name	Plant Part Used	Mode of Use	Reference
*Aconitum rotundifolium*	Whole plant	Plant juice is taken orally along with an equal volume of water for five to seven days to cure jaundice.	[31]
*Adiantum capillus*	Leaves	A decoction of fresh leaves is taken two times for seven days to cure jaundice.	[24]
*Aegle marmelos*	Leaves and fruit	A decoction of leaves and unripe fruit is used to treat jaundice.	[24]
*Ajania tibetica*	Leaves and flower	Leaves and flowers are used to cure jaundice.	[28]
*Aloe vera*	Fleshy leaves	The pulp of the leaves is directly consumed by the patient for two weeks to cure jaundice.	[23]
*Argemone mexicana*	Whole plant	Yellow sap of the plant is used to treat jaundice.	[24]
*Asparagus adscendens*	Roots	A decoction of roots (10–15 mL) is given for eight to ten days to cure jaundice.	[23]
*Aquilegia fragrans*	Seeds	A decoction of seeds is used to treat jaundice.	[34]
*Bauhinia variegata*	Leaves	Leaves juice is taken for seven days for the treatment of jaundice.	[23]
*Berberis aristata*	New leaves (twigs) and roots	New leaves are directly consumed, and a decoction of ground roots (100 mL) is taken to cure jaundice.	[23]
*Berberis ceratophylla*	Roots	Fresh roots are cut into small pieces and further shade-dried to make pills. These pills are consumed with “Kujja-Mishri” with water to cure jaundice.	[34]
*Berberis chitria*	Roots	A decoction of roots is used to treat jaundice.	[27]
*Berberis lycium*	Roots	A decoction of roots (80–100 mL) is given to cure jaundice.	[23]
*Betula utilis*	Papery bark	A decoction of the bark is given to the patient for ten to twelve days to cure jaundice.	[23]
*Boerhavia diffusa*	Whole plant	The whole plant is used to cure jaundice.	[39]
*Cajanus cajan*	Leaves	Leaf juice or leaf decoction is given with sugar (regularly in the morning) for about one month to cure jaundice.	[40]
*Calotropis procera*	Flowers	Flowers and betel leaf are taken with honey to treat jaundice.	[26]
*Capparis spinosa*	Shoot	Stem powder is taken with water at least for five to six days.	[31]
*Capsella bursa-pastoris*	Stem	The stem is used for the treatment of jaundice.	[29]
*Capsicum annuum*	Leaves	Boiled leaves are used as a vegetable (saag) and given for two to three days to cure jaundice.	[23]
*Carica papaya*	Raw fruit	Boiled vegetable of raw fruit is given to the patient to cure jaundice.	[23]
*Carissa opaca*	Roots	Roots are used for the treatment of jaundice.	[25]
*Cassia fistula*	Seeds	A decoction of seeds is consumed empty stomach for a week, daily in the morning.	[34]
*Centella asiatica*	Whole plant (entire herb)	The dried herb is crushed with kali mirch, and its paste (5–10 g is taken for seven days to cure jaundice.	[23]
*Cicer microphyllum*	Seeds	Seeds are used for the treatment of jaundice.	[28]
*Colocacia antiquorum*	Corm	Corm (cooked or pealed) is kept in open places overnight. In the morning, chopped pieces are given with honey to the patient for five days.	[23]
*Colocacia esculenta*	Bulb	The dried bulb in the powder form is used for the treatment of jaundice.	[24]
*Conyza japonica*	Leaves	Leaf paste is used to cure jaundice.	[29]
*Cucumis sativus*	Fruit	Fresh fruit is cut into small pieces and taken thrice a day for three weeks to cure jaundice.	[24]
*Cuscuta reflexa*	Whole plant	A decoction of the whole plant is used to treat jaundice.	[24]
*Crepis flexuosa*	Whole plant	Whole plant juice is mixed with water in equal proportion and taken once a day to cure jaundice.	[31]
*Datura stramonium*	Leaves and fruit	Fruits and leaves are used to cure jaundice.	[42]
*Emblica officinalis*	Roots	A decoction of roots is recommended for two weeks to cure jaundice.	[24]
*Elaeagnus rhamnoides*	Fruit	Fruit juice is used to cure jaundice.	[31]
*Euphorbia hirta*	Stem and leaves	Stem and leaf extract is used to cure jaundice.	[24]
*Euphorbia tirucalli*	Leaves	A decoction of leaves is used for the treatment of jaundice.	[39]
*Equisetum arvense*	Young branch	Young branches are dipped in the water overnight, and juice is (mix a small quantity of Kujja-Mishri and two and a half kali mirch seeds) taken daily empty stomach at least for seven days to cure jaundice.	[23]
*Flacourtia ramontchi*	Bark, fruits, and roots	Bark, fruits, and roots are used to treat jaundice.	[25]
*Gentiana kurrroo*	Roots	The root powder is used for treating jaundice.	[27]
*Gentiana tubiflora*	Whole plant	The whole plant is ground with lazi (salted curd) to form a paste and given for forty to forty-five days to cure jaundice.	[30]
*Gentiana leucomelaena* *Gentianopsis detonsa* *Gentianopsis paludosa*	Whole plant	Plants are crushed with a small proportion of petals of *Polemonium caerulem,* and this mixture is given with curd or cow milk empty stomach for fifteen to twenty-two days to cure jaundice.	[30]
*Gentianella moorcroftiana*	Aerial plant part	Juice of fresh extracted aerial plant part is taken empty stomach to cure jaundice.	[31]
*Gernaium nepalenses*	Roots	Root powder (2 g) is administered thrice a day to cure jaundice.	[42]
*Geum elatum Wallich*	Leaves	Leaves extract, mixed with cow milk or curd, is given for fifteen to twenty-two days to cure jaundice.	[30]
*Hedera helix*	Leaves	Crushed leaves’ juice is used to cure jaundice.	[27]
*Hippophae tibetana*	Fruit	A decoction of the fruit is taken to cure jaundice.	[31]
*Hordeum vulgare*	Seeds	Dried seed powder is mixed with a sugar solution to cure jaundice.	[37]
*Justicia adhatoda*	Roots	A decoction of its roots is given to the patient for one month to cure jaundice.	[24]
*Leucas cephalotes*	Entire herb	Juice of the entire herb (10–15 mL) is given to the patient for eight to ten days.	[23,36]
*Mallotus philippinensis*	Seeds	Seed powder is given for the treatment of jaundice.	
*Mentha spicata*	Leaves	Fresh leaf juice is taken with Kujja-Mishri and given twice a day for two or more weeks.	[23]
*Morus alba*	Fruit	Fruit juice is used for treating jaundice.	[24]
*Morus nigra*	Fruit	Fresh fruit juice is given to the patients twice a day for two weeks.	[24]
*Oroxylum indicum*	Bark of the stem	Crushed bark is soaked in water overnight and given with a small amount of kapoor to cure jaundice. A decoction of the bark is also used for treating jaundice.	[23]
*Persicaria amplexicaulis*	Whole plant	A decoction of the whole plant is given orally to treat jaundice.	[26]
*Picris hieracioides*	Whole plant	Plant extract with salted curd is given to the patient for twenty to thirty days.	[30]
*Pistacia integerrima*	Fruit	Fresh fruit juice is given daily for seven days to curing jaundice.	[24]
*Phyllanthus fraternus*	Whole plant, roots	A decoction of the entire herb and juice of fresh roots is given for seven days to cure jaundice.	[23]
*Picrorhiza kurroa*	Rhizome	Rhizome powder is used to cure jaundice.	[24]
*Polygonum tortuosum*	Whole plant	A paste of the whole plant is mixed with curd (prepared from goat’s milk) and given an empty stomach for fifteen to twenty-two days.	[30]
*Podophyllum hexandrum*	Flower and leaves	The juice of flowers and leaves is mixed with butter and taken orally to cure jaundice.	[41]
*Prunus domestica*	Fruit	The fruit extract is used for the treatment of jaundice.	[24]
*Punica granatum*	Fruit and seeds	Seeds and fruit powder is taken with water and sugar solution to cure jaundice.	[24]
*Raphanus sativus*	Root and fleshy part	A decoction of roots and juice of fleshy part is given to cure jaundice.	[24]
*Ricinus communis*	Leaves	Leaf juice is given with cow’s milk early in the morning for seven days to treat jaundice.	[23]
*Rosa webbiana*	Fruit	Fruit powder is mixed with little quantity of water and taken daily to cure jaundice.	[31]
*Rubia manjith*	Roots and stem	Roots and stem paste is given to cure jaundice.	[42]
*Saccharum officinarum*	Stem	Stem juice is used to cure jaundice.	[24]
*Saxifraga flagellaris*	Leaves and stem	Leaves and stems are used to cure jaundice.	[28]
*Scorzonera divaricata*	Leaves and shoot	A decoction of leaves and shoots is taken orally to cure jaundice.	[31]
*Sesamum indicum*	Leaves	Powder made from fresh leaves is used to cure jaundice.	[24]
*Solanum nigrum*	Leaves	Tablets are made from crushed leaves and taken with imli (tamarind) or curd for treating jaundice.	[23]
*Solanum surattense*	Fruit	The fruit is directly consumed for the treatment of jaundice.	[24]
*Tamarindus indica*	Fruit and root	A decoction of its roots is used to treat jaundice. The fruit is also used to cure jaundice.	[24]
*Taraxacum officinale*	Root and leaves and whole herb	The entire herb in the crushed form (10 gm) is given to the patient for ten days to curing jaundice.	[23]
*Terminalia bellirica*	Leaves	A decoction of leaf powder is taken to cure jaundice.	[36]
*Terminalia chebula*	Fruit rind	Fruit powder is mixed with rock salt and taken with warm water for eight to ten days to cure jaundice.	[23]
*Thalictrum foliolosum*	Roots	A decoction of roots is used to treat jaundice.	[27]
*Tinospora cordifolia*	Fresh stem	The dried stem of the giloe is crushed with punarnava mool, and its juice is taken for seven to ten days.	[23]
*Trigonella emodi*	Leaves and flower	Leaves and flower powder is taken with water twice a day for seven to ten days.	[31]
*Urtica dioica*	Whole plant	The whole plant is used to treat jaundice.	[42]
*Vernonia anthelmintica*	Seeds and leaves	A decoction of seeds and leaves is given to cure jaundice.	[27]
*Viola serpens*	Whole plant	A decoction of dried plant is taken with sugar for more than fifteen days.	[24]
*Woodfordia fruticosa*	Flowers	Flower extract is used to cure jaundice.	[24]
*Youngia tenuifolia*	Leaves	Leaves are used to treat jaundice.	[28]

**Table 3:** Major phytochemicals present in ethnomedicinal plants used for curing jaundice in Himachal Pradesh.

Plant Name	Phytochemical Constituent	Data Source
*Aconitum rotundifolium*	Diterpenoid alkaloids (isoatisine, atisine chloride)	[52]
*Adiantum capillus-veneris*	Flavonoids, phenolic acids (sulfate esters of hydroxycinnamic acid), alkaloids, terpenoids (triterpenes), steroids, tannins, saponins	[53]
*Aegle marmelos*	Phenolic compounds (coumarins, such as marmelosin, marmesin, imperatorin, scopoletin, and esculetin), alkaloids (aeglin, aegelenine, skimmianine), tannins	[54]
*Ajania tibetica*	Terpenoids (bornyl acetate 60.7%, β-caryophyllene 9.1%, β-eudesmol 5.3%, methyl thymol 4.3% and borneol 2.2%)	[55]
*Aloe vera*	Fatty acids (such as n-hexadecanoic acid 20.47%, oleic acid 14.53%, tetradecanoic acid 1.04%, 1,2-benzenedicarboxylic acid, diisooctyl ester 13.60%, squalene 6.60%, butyl octyl ester 2.30%)	[56]
*Argemone mexicana*	Alkaloids (argemexicaine A, argemexicaine B, protopine, columbamin, muramine, cryptopine, isocorydine), carbohydrates (arabinose, lactose), steroids (β-sitosterol, stigma-4-en-3,6-dione), terpenoids (β-amyrin, trans-phytol), flavonoids (eriodictyol, luteolin, quercetin, rutin), tannins, phenolic acids (vanillic acid)	[57]
*Asparagus adscendens*	Saponins and steroids (stigmasterol glycosides)	[26]
*Aquilegia fragrans*	Steroids (β-sitosterol), 2,4-dihydroxyphenylacetic acid methyl ester, aquilegiolide, glochidiono lactone-A, and alkaloids (magnoflorine)	[58]
*Bauhinia variegata*	Terpenoids, flavonoids, tannins, saponins, steroids, and cardiac glycosides	[26]
*Berberis aristata*	Phenolic acids (e-caffeic acid, chlorogenic acid), flavonoids (quercetin, rutin), and alkaloids (berberine, berbamine, palmatine, columbamine, jatrorrhizine, oxyacanthine)	[59]
*Berberis lycium*	Alkaloids (berberine, berbamine, chenabine, karakoramine, palmatine, baluchistanamine, gilgitine, jhelumine, punjabine, sindamine)	[26]
*Betula utilis*	Terpenoids (betulin, betulinic acid, oleanolic acid, acetyl-oleanolic acid, lupeol, lupenone, methyl betulonate, methyl betulate, karachic acid), steroids (sitosterol), flavonoids (leucocyanidin, polymeric leucoanthocyanidins)	[60]
*Boerhavia diffusa*	Flavonoids (rotenoids, quercetin, kaempferol, borhaavone), lignans, steroids, phenolic glycosides, phenolic compounds (trans-caftaric acid, xanthones), fatty acids, and hydrocarbons	[61]
*Cajanus cajan*	Carbohydrates 26.425 ± 0.32, proteins 12.83 ± 0.285, lipids and phenols	[62]
*Calotropis procera*	Alkaloids (calotropin, calotoxin, uskerin), flavonoids, tannins, saponins, cardiac glycosides, volatile oil, and steroids	[26]
*Capparis spinosa*	Flavonoids (flavonol, quercetin-7-O-β-d-glucopyranoside-β-l-rhamnopyranoside, quercetin-3-rutinoside, rutin), fatty oil, carbohydrates (pentosans), and saponin	[31]
*Capsella bursa-pastoris*	Fatty acids (dodecanoic acid 5.66 ± 1.17, tetradecanoic acid 29.63 ± 5.79, pentadecanoic acid 18.05 ± 3.06, hexadecanoic acid 284.48 ± 41.06, heptadecanoic acid 7.11 ± 1.60, octadecanoic acid 53.20 ± 0.68, eicosanoic acid 2.52 ± 0.33), steroids (phytosterol, cholesterol, campesterol, stigmasterol, β-sitosterol), amino acids (glycine, histidine), and flavonoids (tricin, kaempferol, quercetin)	[63]
*Capsicum annum*	Alkaloids (capsaicin 0.5%–0.9%), glycosides, carbohydrates, steroids, terpenoids (triterpenes), and carotenoids (capsanthin, capsorubin 4–16%)	[64]
*Carica papaya*	Flavonoids, saponins, tannins, glycosides, and steroids	[65]
*Carissa opaca*	Cardiac glycosides (digitoxigenin-3-O-β-d-digitalopyranoside), phenolic compounds, lignans, terpenoids (17-hydroxy-11-oxo-nor-β-amyrone, urs-12-ene-3β, 22β-diol-17-carboxylic acid), steroids (stigmasterol, campesterol, β-sitosterol), flavonoids (rutin, quercetin), essential oils (hydroxyacetophenone 89.5%, benzyl salicylate 6.0%, benzyl benzoate 4.6%, (E,E)-α-farnesene 3.5%), protein (1.3%), and carbohydrates (17.39%)	[66]
*Cassia fistula*	Terpenoids (lupeol), steroids (β-sitosterol), fatty acid alcohols (hexacosanol), flavonoids (kaempferol, leucopelargonidin, rhamnetin-3-O-gentiobioside), phenolic acids (rhein, 3-formyl-1-hydroxy-8- methoxy anthaquinone), and alkaloids	[26]
*Centella asiatica*	Alkaloids, glycosides, terpenoids, steroids, flavonoids, tannins, and reducing sugars	[67]
*Cicer microphyllum*	Steroids (phytosterols), flavonoids, phenolic compounds, tannins, carbohydrates, proteins, and amino acids	[68]
*Colocasia esculenta*	Flavonoids (flavones, apigenin, luteolin, anthocyanins), carbohydrate (starch 0.23–0.52%), and lipids 0.017–0.025%	[69]
*Conyza japonica*	Terpenoids (sesquiterpenoids, conyterpenols A−D, strictic acids), flavonoids, and their glycosides	[70]
*Crepis flexuosa*	Phenolic acids (p-hydroxybenzoic acid, ethyl p-hydroxybenzoate, esculetin) terpenoids (taraxast-20(30)-ene-3β, 21α-diol, ursolic acid, oleanolic acid), flavonoids (apigenin, luteolin, luteolin-7-O-β-D-glucoside), fatty acids (octacosanoic acid, 2′,3′-dihydroxypropyl pentacosanoate), and steroids (daucosterol)	[71]
*Cuscuta reflexa*	Flavonoids (aromandendrin), glycosides, carotenoids (lutein- 10–22%, lycopene), alkaloids, steroids (campesterol, stigmasterol, stigmast-5-en-3-O-β-D-glucopyranoside), lignin (sesamin), and terpenoids (lupeol, maragenin)	[72]
*Cucumis sativus*	Steroids, glycosides, flavonoids, alkaloids, saponins, and tannins (except gums and reducing sugars)	[73]
*Datura stramonium*	Alkaloids (atropine, hyoscyamine, scopolamine), glycosides, saponins, and tannins	[74]
*Elaeagnus rhamnoides*	Alkaloids (carboline), terpenoids (ursolic acid, uvaol, amyrin), flavonoids (quercetin, myricetin, isorhamnetin, glucosides, rutin), carotenoids, fatty oil, and steroids (sitosterol, citrostandienol)	[31]
*Emblica officinalis*	Phenolic acids (propnyl 3,4,5-trihydroxybezonate, 2,3,7,8-tetrahydroxy chromeno [5,4,3-cde]chromene-5,10-dione, chlorogenic acid, ellagic acid), flavonoids (rutin, quercetin), tannins, amino acids, fixed oils	[75]
*Euphorbia hirta*	Terpenoids (diterpenes, triterpenes), phenolic acids (coumarins), and lignans	[76]
*Euphorbia tirucalli*	Fatty acids (palmitic acid, linoleic acid), steroids (sitosterol, stigmasterol, campesterol), flavonoids (anthocyanin, cyanidin glycoside)	[77]
*Equisetum arvense*	Flavonoids 0.6–0.9% (such as apigenin-5-O-glucoside, genkwanin-5-O-glucoside, kaempferol-3,7-di-O-glucoside, kaempferol-3-O-(6′-O-malonylglucoside)-7-O-glucoside, kaempferol-3-O-sophoroside, luteolin-5-O-glucoside, quercetin-3-O-glucoside), terpenoids (cis-geranyl acetone 13.74%, thymol 12.09%, trans-phytol 10.06%, triterpenes), alkaloids, carbohydrates, proteins, amino acids, steroids (phytosterols), saponins, and tannins	[78]
*Flacourtia ramontchi*	Saponins, steroids, sugar, lignans, and terpenoids (triterpenes)	[79]
*Geranium nepalenses*	Steroids (β-sitosterol, β-sitosterol-glactoside, stigmasterol) and terpenoids (ursolic acid)	[80]
*Gentiana kurroo*	Terpenoids (iridoids, triterpenoids), flavonoids, alkaloids	[81]
*Gentianopsis paludosa*	Flavonoids (luteolin), xanthones (gentiacaulein, phenolics-1-hydroxy-3,7,8-trimethoxyxanthone), terpenoids (ursolic acid), dicarboxylic acid–succinic acid	[31]
*Gentianopsis detonsa*	Flavonoids (luteolin), dicarboxylic acid–succinic acid, xanthones (gentiacaulein, 1-hydroxy-3,7,8-trimethoxyxanthone), terpenoids (ursolic acid)	[31]
*Geum elatum*	Fatty acid alcohols (hentriacontanol, hentriacontanone), sterols (β-sitosterol), phenolic acids (tetra-O-methyl ellagic acid, ellagic acid), and flavonoids (isoquercetrin)	[82]
*Hedera helix*	Phenolic acids (gallic acid 131.25 ± 1.54), flavonoids (quercetin 18.61 ± 0.37), alkaloids (emetine), amino acids, and saponins (hederacoside C, α-hederin, hederagenin)	[83]
*Hippophae tibetana*	Flavonoids (isorhamnetin, quercetin, kaempferol, rhamnetin, quercetin -3-O- rutinoside, quercetin-3-O-galactoside), fatty acids (2-hydroxydecanoic acid, nona-7-enoic acid, undec-9-en-7-ynoic acid, 13-phenyl tridecanoic acid, 5,9,21-nonacosatrienoic acid, 1,3-dicapryloyl-2- linoleoylglycerol, oleic, linoleic, linolenic acids and fats 3.5–4.8%)	[84]
*Hordeum vulgare*	Flavonoids, phenolic acids, terpenoids, glycosides, and saponins	[85]
*Justicia adhatoda*	Alkaloids (vasicine, vasicol, vasicinone, peganine, adhatonine, vasicinol, vasicinolone), flavonoids (kaempferol, quercetin), and steroids (β-sitosterol)	[26,86]
*Leucas cephalotes*	Phenolic acids (gallic acid, protocatechuic acid, chlorogenic acid, caffeic acid, and ferulic acid)	[26]
*Mentha spicata*	Fatty acids (methyl esters, methyl acetate 2–11%), terpenoids (menthol 33–60%, menthone 15–32%, isomenthone 2–8%, 1,8 cineole eucalyptol 5–13%, menthofuaran 1–10%, limonene 1–7%), flavonoids, alkaloids, and sugars	[87]
*Morus alba*	Phenolic acids (coumarins, benzofurans), flavonoids (chalcones, flavones, flavane derivative, (2S)-4′-hydroxy-7-methoxy-8-prenylflavan)	[88]
*Morus nigra*	Phenolic acids, alkaloids, terpenoids (oleanolic acid), and flavonoids (quercetin, luteolin, apigenin, kuwanon, kaempferol)	[89]
*Oroxylum indicum*	Steroids, tannins, alkaloids, glycosides, and flavonoids	[90]
*Persicaria amplexicaulis*	Flavonoids (quercetin), steroids (β-sitosterol), phenolic compounds (methyl-4-hydroxy cinnamate, gallic acid, protocatechuic acid, methyl gallate, vanicoside A, vanicoside B), terpenoids (arborinone), 25-hydroxycholest-5-en3β-yl acetate	[91]
*Phyllanthus niruri*	Alkaloids, terpenoids (triterpenes), phenols, flavonoids (quercetin, kaempferol), lignin glycoside, tannins, and fatty acids	[92]
*Picrorrhiza kurroa*	Terpenoids (iridoid glycoside, triterpenoids, kutkin, picroside I 3.66 ± 0.11%, kutkoside 4.44 ± 0.02%), steroids, tannins, and saponins	[93]
*Picris hieracioides*	Terpenoids (gammacer-16-ene derivatives, gammacer-16-en-3β-yl acetate)	[94]
*Pistacia integerrima*	Phenolic acids, carotenoids, terpenoids (monoterpens 91%, triterpenes), flavonoids (catechins), saponins, tannins, and steroids	[95]
*Podophyllum hexandrum*	Terpenoids, steroids, flavonoids, saponins, tannins, glycosides, and amino acids	[96]
*Punica granatum*	Flavonoids, saponins, tannins, phenolic acids, glycosides, steroids (phytosterols), terpenoids, carbohydrates, and proteins	[97]
*Prunus domestica*	Flavonoids, phenylpropanoid esters, phenolic acids (caffeoylquinic acids), steroids, and terpenoids	[98]
*Raphanus sativus*	Tannins (phlobatannins), saponins, flavonoids, phenolic acids (anthraquinones), steroids (phytosterol), alkaloids, terpenoids, cardiac glycosides, glucosinolates, isothiocyanates, protein 28.57%, carbohydrates 39.82% and fats 27.76%	[99]
*Ricinus communis*	Alkaloids, steroids, glycosides, flavonoids (quercetin, vitexin, rutin, kaempferol, epicatechin), terpenoids, phenolic acids (gentisic acid, ellagic acid, gallic acid, coumarins), and essential oils 37%	[100]
*Rosa webbiana*	Flavonoids, alkaloids, tannins, and saponins	[101]
*Rubia manjith*	Phenolic acids (quinones like glycosides, including rubiadin,1-hydroxy,2-methoxy anthraquinone, 3-dimethoxy 2 carboxy anthraquinone, munjistin, purpurin, pseudopurpurin, mollugin, furomollugin), fatty acids (rubiprasin A,B,C), ruiearbonls, and terpenoids (aborane, triterpenes)	[102]
*Saccharum officinarum*	Steroids (phytosterols), terpenoids, flavonoids, -O- and -C-glycosides, phenolic acids, fatty acid alcohols (policosanoles 2.5–80%, octacosanol 50–80% of the total policosanoles)	[103]
*Sesamum indicum*	Phenolic acids (anthraquinone) and tannins	[104]
*Solanum nigrum*	Flavonoids (catechin, epicatechin, rutin), phenolic acids (caffeic acid, gallic acid, protocatechuic acid), fatty acids (linoleic acid 67.9%), carbohydrates (polysaccharides), and proteins content 17%	[105]
*Solanum surattense*	Alkaloids, tannins, saponins, phenolic acids (phenolic methyl caffeate, caffeic acid, coumarins like imperatorin, scopoletin and esculetin), steroids (β-sitosterol), tri-terpenoids, and other major constituents like solasonine, solamargine, solasurine, torvoside K and L, khasianine, aculeatiside A, and solamargine	[106]
*Taraxacum officinale*	Free amino acids, terpenoids (germacranolide, taraxacin, taraxacerin, a diester of taraxanthin, lactupicrin, triterpenes), carbohydrates (glucans, mannan), phenolic acids (scopoletin, esculetin), steroids (phytosterols, taraxasterol, homotaraxasterol), eudesmanolic-tetrahydroridentin B, eudesmanolide-d-glucopyranoside, and proteins	[31]
*Tamarindus indica*	Fatty acids (n-heptadecanoate 13.00%, n-octadecanoic 6.1%, methyl-n-pentacosanoic 4.45%, nonanoic acid 1.92%, nonadecanoic acid 9.2%, 10-octadecenoicacid 7.8%, heptadecanoate 3.3%, n-pentacosenoic acid 2.54%, hexacoseoic acid 0.7%) and proteins 7.5–6.6%	[107]
*Terminalia bellirica*	Fatty acids (stearic acid 14.93%, myristic acid 17.70%, palmitic acid 21.6%, oleic acid 45.67%), proteins, carbohydrates, steroids (β-sitosterol), tannins (chebulanic acid, galloyl glucose), phenolic acids (gallic, ellagic acid, ethyl gallate), alkaloids, flavonoids, saponins, and terpenoids	[108]
*Terminalia chebula*	Tannins (chebulic acid, chebulagic acid, corilagin), phenolic acids (gallic acid, ellagic acid), steroids (β-sitosterol), terpenoids (triterpenes), and flavonoids (flavonol glycosides)	[109]
*Thalictrum foliolosum*	Alkaloids (berberine, jatrorrhizine, palmatine, thalrugosidine, thalrugosaminine, thalisopine thaligosine, thalirugidine, trhalirugine, 8-oxyberberine, berlambine, noroxyhydrastinine, N, O, O-trimethylsparsiflorine, thalicarpine, thalidasine, thalfoliolosumines A, and thalfoliolosumines B)	[110]
*Tinospora cordifolia*	Phenolic acids, flavonoids, glycosides, saponins, and alkaloids	[111]
*Trigonella emodi* Ben	Flavonoids (quercetin, luteolin, vitexin, orientin, isoorientin, vicenin-1, vicenin-2, naringenin, kaempferol, 7,4′-dimethoxyflavanone), protein, and carbohydrates	[112]
*Urtica dioica*	Alkaloids (betaine, choline), amino acids, carbohydrates, protein polymer (neutral and acidic), carotenoids (carotenes), and saponins	[31]
*Vernonia anthelmintica*	Fatty acids (vernolic acid) and terpenoids (vernodalin, vernodalol)	[113]
*Viola serpens*	Tannins, amino acids, reducing sugars, flavonoids (rutin),organic ester (methyl salicylate), glycosides (quercitrin), alkaloids (violin), terpenoids (monoterpens, sesquiterpenes), saponin, bis (2-ethylhexyl) maleate 15.62%, 2,4,4,6-tetramethyl-2-heptene 11.52%, hexen-3-ol 6.56% and cis verbeno l 4.77%	[114,115]
*Woodfordia fruticosa*	Phenolic acids, tannins (hydrolyzable tannins, such as woodfordins A, B, C), flavonoids (quercetin glycosides, naringenin 7-glucoside, kaempferol 3-O-glucoside), fatty acid alcohols (octacosanol), steroids (β-sitosterol, hecogenin), and terpenoids (lupeol, betulin, ursolic acid, oleanolic acid)	[116]

**Table 4:** In vivo evidence of ethnomedicinal plants used for the treatment of jaundice/hepatoprotective activity.

Plant Species	Plant Part Used (Extract Taken/Total Amount or Dose Required)	Test Dose/ Experimental Model	Constituents Responsible for (May/May Not Be Present)	Data Source
*Aegle marmelos*	Pulp/seeds (aq. extract/NA)	CCl_4_-induced hepatotoxicity/albino Wistar rat	NA	[15]
	Powdered fruit pulp	Gentamicin-induced liver injury/oral/Wistar albino rats	NA	
	Leaves/powder	Ethanol-induced liver toxicity/orally/male albino Wistar rat	NA	
*Aloe vera*	Aerial part (aq. extract/500 mg/kg bw)	CCl_4_ (1 mL/kg)/albino Wistar rat	NA	[5]
*Argemone mexicana*	Stem (aq. extract/250 and 150 mg/kg bw)	CCl_4_-induced hepatotoxicity (2 mL/kg bw)/albino Wistar rat	NA	[5]
*Bauhinia variegata*	Stem/bark extract/100 and 200 mg/kg)	CCl_4_ (1 mL/kg)/Sprague-Dawley rats	NA	[119]
*Berberis aristata*	Root extract/NA)	CCl_4_ (1 mL/kg)/albino rats	Berberine	[120]
*Berberis chitria*	Bark, stem extract/80 mg Livshis sample (450 mg)	CCl_4_ (1 mL/kg)/male Wistar albino rats	NA	[121]
*Boerhavia diffusa*	Root (aq. extract/2 mL/kg and 150 mg/kg bw)	Thioacetamide (100 mg/kg bw)/albino Wistar rat	Ursolic acids	[5]
*Cajanus cajan*	Leaves (methanolic extract/100 mg/kg bw)	Acetaminophen and D-galactosamine-induced hepatotoxicity/albino Wistar rat	Alkaloids and flavonoids	[15]
*Calotropis procera*	Flowers (hydro-ethanolic extract/200 mg/kg and 400 mg/kg)	Paracetamol (2 g/kg)/Wistar rats	Quercetin-3-rutinoside and other flavonoids	[122]
*Capparis spinosa*	Root bark (ethanolic extract/100, 200, and 400 mg/kg)	CCl_4_ (0.2 mg/kg)/mice	NA	[123]
*Carica papaya*	Seeds (aq. extract/(400 mg/kg)	CCl_4_ (1.5 mL/kg)/male Wistar rat	NA	[124]
*Carissa opaca*	Leaves (methanolic extract/(200 mg/kg bw)	CCl4 (0.5 mL/kg)/Sprague-Dawley rats	Isoquercetin, hyperoside, vitexin, myricetin, and kaempferol	[125]
*Cassia fistula*	Fruit pulp (aq. extract 2000 mg/kg bw)	CCl_4_ (1 mL/kg bw)/albino Wistar rat	Lupenol	[5]
*Centella asiatica*	Whole plant (aq. extract/(0.7 g/kg bw))	CCl_4_ (0.7 mL/kg bw)/albino Wistar rat	NA	[5]
*Cuscuta reflexa*	Whole plant (hydro- alcoholic extract/400 mg/kg bw)	Paracetamol (200 mg/kg bw)/albino Wistar rat	Phenolic compounds	[5]
*Colocasia antiquorum*	Corms (petroleum ether extract/NA)	Paracetamol-induced hepatotoxicity (100 mg/kg bw)/albino mice	Anthocyanins	[126]
*Euphorbia hirta*	Whole plant (aq. extract/100–300 mg/kg bw)	CCl_4_-induced hepatotoxicity (NA)/adult male Wistar rat	Flavonoids	[127]
*Euphorbia tirucalli*	Arial parts (aq. extract/125–200 mg/kg bw)	CCI_4_ intoxicated (NA)/albino Wistar rat	Flavonoids	[128]
*Hedera helix*	Leaf (aq. extract/150 mg/kg bw)	CCl_4_-induced hepatotoxicity (5 mL/kg)/	NA	[129]
*Hordeum vulgare*	Seeds (methanolic extract/300–500 mg/kg)	ethanol-induced liver damage (3.76 g/kg/day)/Wistar albino rat	Phenolic compounds	[85]
*Justicia adhatoda*	Leaves and flowers (methanolic extract/200 mg/kg bw)	CCl_4_-induced hepatotoxicity (1 mL/kg of body bw)/Swiss albino mice	NA	[130]
*Flacourtia ramontchi*	Leaf (aq. extract/250 and 500 mg/kg bw)	CCl_4_ (1.5 mL/kg bw)/albino Wistar rat	Phenolic compounds	[5]
*Leucas cephalotes*	Whole plant (methanolic extract/400 mg/kg bw)	CCl_4_-induced liver toxicity (NA)/male mice	Flavonoid	[15]
*Morus alba*	Leaves (petroleum ether chloroform alcoholic and water extract 200–500 mg/kg)	CCl_4_-induced hepatotoxicity (1 mL/kg)/Swiss albino mice	Alkaloids, carbohydrates, flavonoids, tannins, steroid	[131]
*Morus nigra*	Leaves (aq. methanolic extract/200 and 500 mg/kg)	Paracetamol-induced hepatotoxicity (NA)/mice	Quercetin, luteolin, and isorhamnetin	[132]
*Oroxylum indicum*	Leaves (ethanol, water, chloroform, and petroleum ether/300 mg/kg)	CCl_4_ hepatoprotective activity (0.7 mL/kg)/adult albino rats	Flavonoids and phenolics	[133]
*Phyllanthus emblica*	Fruit (ethanol extract/75 mg/kg/day bw)	Ethanol induced (4 g/kg/day bw)/albino Wistar rat	Phyllanthin and hypophyllanthin	[5]
*Phyllanthus niruri*	Leaves (aq. extract/100 mg/kg bw)	CCl_4_-induced hepatotoxicity (1 mL/kg body weight))/male adult rats	Phenolic compounds, Phyllanthin, and hypophyllanthin	[134]
*Podophyllum hexandrum*	(Hexane extract/50 mg/kg)	CCl_4_-induced hepatotoxicity (NA)/male albino rats	Polyphenols	[135]
*Prunus domestica*	Fruit (methanolic and ethanolic extract/20–100 mg/kg)	Paracetamol-induced hepatotoxicity (2 g/kg)/albino rats	NA	[136]
*Punica granatum*	Leaf (aq. extract/NA)	CCl_4_-induced hepatotoxicity (NA)/albino rats	Flavonoids	[137]
*Raphanus sativus*	Leaves (methanolic extract/300 mg/kg bw)	CCl_4_-induced cytotoxicity/male rats	NA	[15]
*Ricinus communis*	Leaves (ethanolic extract/800 mg/kg)	Paracetamol-induced cholestasis (2 g/kg)/adult Druckrey rats	Ricinine and n-demethyl-ricinine	[138]
*Solanum nigrum*	Whole plant (water or methanolic extract 500 mg/kg)	CCl_4_-induced hepatotoxicity (0.2 mg/kg)/Wistar albino rat	NA	[139]
*Tamarindus Indica*	Stem bark (ethanolic extract/NA)	Hepatic damage induced (100 to 200 mg/kg bw)/female SD rats	NA	[140]
*Terminalia chebula*	Fruit (aq. extract/NA)	t-BHP-induced hepatotoxicity/(NA)/mice	Phenolic compounds	[47]
*Thalictrum foliolosum*	Roots (ethanolic extract (200 to 1000 mg/kg bw))	Paracetamol-induced hepatotoxicity (2 g/kg bw)/Wistar rat and male albino mice	NA	[141]
*Tinospora cordifolia*	Root, stem (petroleum ether/ethanol and aq. extract/400 mg/kg bw)	CCl_4_-induced	Flavonoids, alkaloids, and phenolics	[15]
		liver toxicity/albino Wistar rat		
*Urtica dioica*	Seeds (polar extract/NA)	CCl_4_-induced	Phenolics	[142]
		hepatotoxicity (150 to 200 g/kg)/male Wistar albino rat		
*Woodfordia fruticosa*	Flower (petroleum ether, chloroform, ethanolic/250 mg/kg bw)	CCl_4_ (1%)/albino Wistar rat	NA	[5]
